# Post-translational modifications and their implications in cancer

**DOI:** 10.3389/fonc.2023.1240115

**Published:** 2023-09-19

**Authors:** Hashnu Dutta, Nishant Jain

**Affiliations:** ^1^ Department of Applied Biology, CSIR-Indian Institute of Chemical Technology, Hyderabad, India; ^2^ Academy of Scientific and Innovative Research (AcSIR), Ghaziabad, India

**Keywords:** post-translational modifications, protein structure, cancer, proteome diversity, protein function, covalent bond

## Abstract

Post-translational modifications (PTMs) are crucial regulatory mechanisms that alter the properties of a protein by covalently attaching a modified chemical group to some of its amino acid residues. PTMs modulate essential physiological processes such as signal transduction, metabolism, protein localization, and turnover and have clinical relevance in cancer and age-related pathologies. Majority of proteins undergo post-translational modifications, irrespective of their occurrence in or after protein biosynthesis. Post-translational modifications link to amino acid termini or side chains, causing the protein backbone to get cleaved, spliced, or cyclized, to name a few. These chemical modifications expand the diversity of the proteome and regulate protein activity, structure, locations, functions, and protein-protein interactions (PPIs). This ability to modify the physical and chemical properties and functions of proteins render PTMs vital. To date, over 200 different protein modifications have been reported, owing to advanced detection technologies. Some of these modifications include phosphorylation, glycosylation, methylation, acetylation, and ubiquitination. Here, we discuss about the existing as well as some novel post-translational protein modifications, with their implications in aberrant states, which will help us better understand the modified sites in different proteins and the effect of PTMs on protein functions in core biological processes and progression in cancer.

## Introduction

Post-translational modifications are typical biochemical reactions that covalently bind (poly)peptide chains, chemical moieties, lipids, or carbohydrates to amino acids of the target molecule during or after its translation (1). PTMs occur in majority of known proteins, and nearly all amino acids can be changed by one or more of these reactions. The modified proteins acquire uncommon amino acids that can have a significant impact on their structure and function (2). Akin to noncovalent binding, PTM processes may occur at the functional site (orthosteric PTMs) namely sumoylation, or at a distance (allosteric PTMs) namely, ubiquitination and phosphorylation (1).

**Figure 1:**
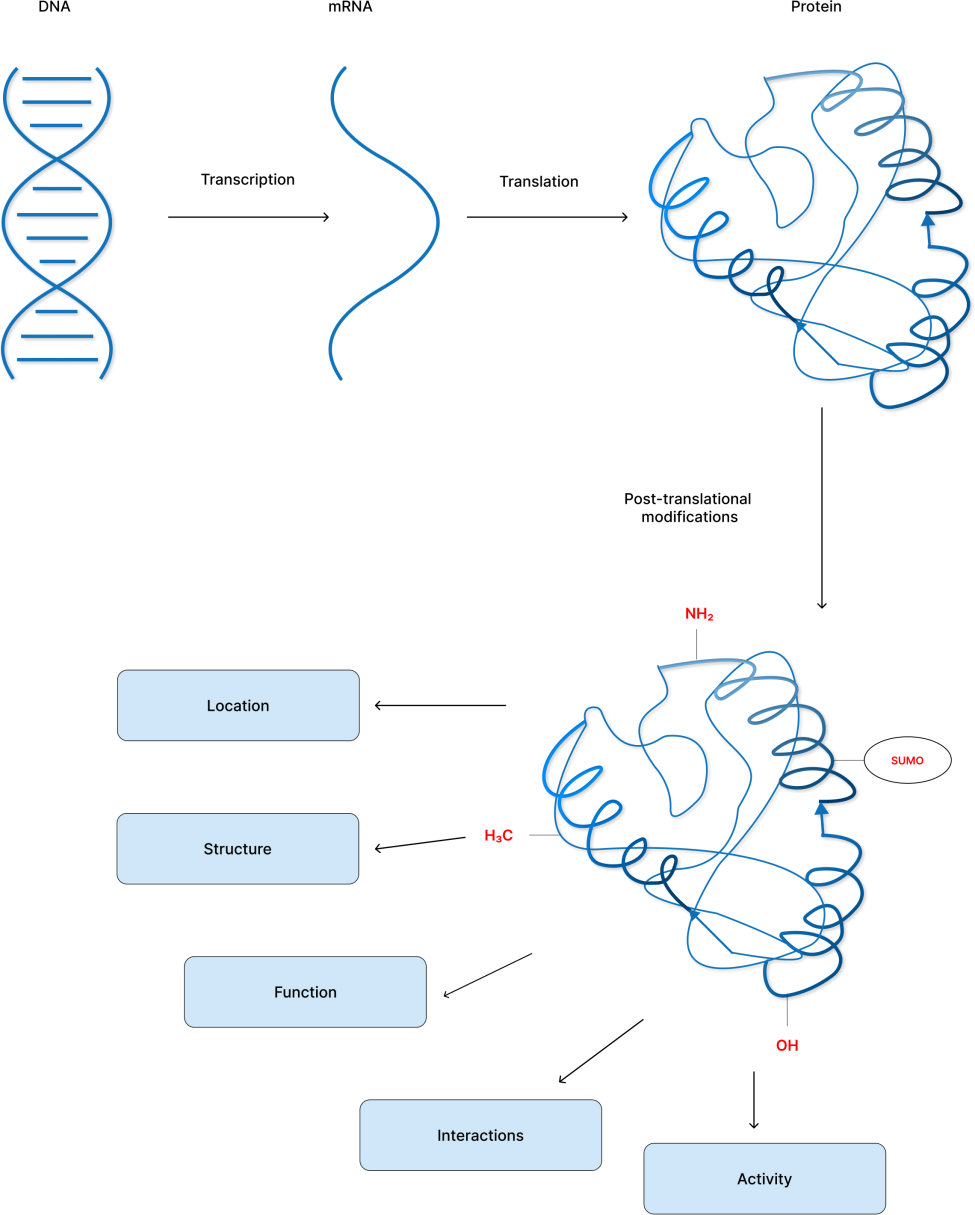
Post-translational modification of proteins increase proteome diversity to perform the core physiological processes.

Post-translational modifications diversify the proteome by altering protein structure, location, interactions, and function and their regulation, thus affecting all facets of cell biology (**Figure 1**) (3). Currently, the UniProt database contains over 400 distinct PTMs that are covalently bonded to amino acid side chains. PTMs range in size from moderate alterations such as methylation to high-molecular-weight polyprotein chains such as polyubiquitination (4, 5).

Protein modifications are commonly catalyzed by enzymes, although a number of reports have stated that some PTMs may happen spontaneous. Non-enzymatic PTMs (that occur spontaneously) can be divided into two subclasses: irreversible and reversible non-enzymatic PTMs, based on their stability and physiological shelf-life. Non-enzymatic irreversible PTMs include stress-induced modifications, such as oxidative and metabolic stresses, that cannot be eliminated by spontaneous dissociation or enzyme-catalyzed mechanisms. These irreversible PTMs occur in a wide spectrum of amino acids, especially when basal levels of reactive oxygen species (ROS) rise and/or when metabolite concentrations approach a physiological imbalance, during aging or in diseases such as cancer, diabetes, and Alzheimer’s disease (6–9). On the contrary, non-enzymatic reversible PTMs are physiologically reversible. The dissociation of these reversible PTMs can be enzyme catalyzed, or from an innate chemical instability, which is involved in various metabolic and signaling pathways.

Eukaryotes exploit post-translational modifications to modulate protein stability, function, subcellular localization, and different protein–protein interactions (10–13). PTMs control protein function of diverse physiological and pathological cellular events. Furthermore, PTMs have been reported to be linked to neurodegenerative disorders, brain tumors, breast neoplasias, and hematological disorders (14, 15). Numerous other forms of PTMs can occur due to metabolic stress. For instance, disruption of Krebs cycle enzymes might increase the fumarate levels followed by succination of cysteine residues by Michael addition reaction (16, 17). Higher succination rates can also be caused by cancer-related mutations influencing fumarate metabolizing proteins, and hence can function as a cancer biomarker (18, 19).

Despite conventional PTMs such as phosphorylation, acetylation, ubiquitination, and methylation that have been extensively studied, the emergence of numerous new modifications, such as hydroxybutyrylation, lactylation, and succinylation, add another dimension to protein regulation, shedding light to many yet-to-be-discovered modifications and their application possibilities per se (20). In this mini-review, we outline a brief overview of both classical (which have been identified within the past few decades) and the novel (newly discovered) post-translational modifications, as well as the important roles these PTMs offer in regulating human cancer hallmarks using relevant examples. Also, the different PTMs that are discussed here have been summarized highlighting the reactions involved and the enzymes catalyzing it (**Table 1**).

**Table 1:** An overview of different post-translational modifications and the enzymes catalyzing these reactions.

Type of Modification	Description	Enzyme(s) involved
Phosphorylation	Addition of a phosphate group to a molecule or an ion	Kinases
Acetylation	Covalent transfer of an acetyl group from acetyl-CoA to the ε-amino group of lysine residues within the target substrates	Acetyltransferases
Glycosylation	Saccharides form glycosidic bridges with other saccharides, proteins, or lipids enzymatically	Glycosyltransferases
Methylation	Addition of a methyl group at both their N- and C-termini, as well as on side chain nitrogen atoms of lysine and arginine residues	Methyltransferases, PRMTs (Lys Methyltransferases and/or Arg Methyltransferases)
Sumoylation	Covalently attachment of small ubiquitin-like modifiers (SUMO) in the ϵ-amino group of lysine residues in the target protein	An E1 activating enzyme, an E2 conjugating enzyme and, an E3 SUMO ligase (most cases)
Ubiquitination	Covalently attachment of Ubiquitin (Ub) to protein substrates	Ub-activating (E1), Ub-conjugation (E2), and Ub-ligase (E3) enzymes
Neddylation	Reversible covalent attachment of NEDD8 to a lysine residue of the protein substrate	NEDD8-activating E1, NEDD8-conjugation E2, and substrate-specific NEDD8-E3 ligase enzymes
S-Nitrosylation	Covalent modification in which a nitric oxide (NO) molecule is coupled to the reactive thiol of an adjacent cysteine residue to generate S-nitrosothiol (SNO)	Nitric oxide (NO) synthases, S-nitrosothiol (SNO) synthases and transnitrosylases
Succination	Stable reaction between fumarate and the thiol groups on cysteine residues to produce S-(2-succino)cysteine (2SC) forming a thioether linkage	Fumarase/Fumarate hydratase (FH)
Prenylation	Attachment of either a 15-carbon farnesyl or a 20-carbon geranylgeranyl isoprenoid lipid to the cysteine residues	Farnesyltransferase (FTase) or Geranylgeranyltransferase I (GGTase I)
Palmitoylation	Reversible lipid modification by which palmitate is added via a thioester linkage to a cysteine residue	Palmitoyl acyltransferases (PATs)
Succinylation	Transfer of a succinyl group to a lysine residue via enzymatic or non-enzymatic reactions, forming an ester, thioester or amide bond	Succinate dehydrogenase (SDH)
Citrullination	Deamination of a positively charged arginine into the electrically neutral citrulline, when subjected to high Ca^2+^ concentration	Peptidyl-arginine deiminase (PAD)
Crotonylation	Evolutionary conserved, epigenetic reversible modification (short chain lysine acylations) on histone and non-histone proteins	Crotonyltransferases (writers) and Decrotonylases (erasers)
S-Glutathionylation	A reversible reaction in which cysteine residue of glutathione forms a disulphide bond with the -SH (thiol) group of a target cysteine in the protein, oxidising it	Glutaredoxins (GRX), thiol oxidoreductases
Monoaminylation	Biogenic monoamines are covalently linked to glutamine residues within certain protein substrates via transamidation reaction	Transglutaminase 2
Propionylation	Covalent attachment of the propionyl group to the lysine residues of the target protein	Propionyl-CoA Carboxylase (PCC); also, p300 and CREB-binding protein (CBP)
Butyrylation	Covalent alteration of butyryl group to the amino acid, lysine	p300 and CREB-binding protein (CBP) as butyryl-modifying enzymes (butyryl transferases) Debutyrylases (sirtuins)
Lactylation	A modification by which L-lactate (a cellular metabolite byproduct) changes lysine residues on histone proteins (Kla or Klac)	YiaC/Lactylase and CobB/Delactylase

## Classical post-translational modifications

### Phosphorylation

Phosphorylation is the most researched and one of the essential types of PTM, which frequently occurs either in nucleus or in cytosol on target proteins in all organisms (21, 22). The kinase enzyme catalyzing the phosphorylation reaction transfers a phosphate group from adenosine triphosphate (ATP) to the receptor residues, thereby altering the protein function in either of the two processes: via allostery or by interacting with the adjoining domains (22). Phosphorylation predominantly occurs on target sites comprising Ser, Thr, Tyr, and His residues (23). In contrast, dephosphorylation or elimination of a phosphate group is a phosphatase-mediated enzymatic reaction (24).

Cancer is one of the most abhorrent disorders in which numerous regulatory gene products are transformed (25). Recent studies have highlighted the involvement of aberrant protein phosphorylation in cancer manifestation (26–29). The primary mechanisms underlying impaired signal transduction are due to uncontrolled phosphorylation (gain- or loss-of -function mutation) (30). Yet, mutations in kinases or phosphatases which induce aberrant phosphorylation and upset the delicate equilibrium of activation or inactivation of cancer-associated proteins are regarded as both the cause and effect of cancer (31).

### Acetylation

Acetylation modification is a reversible acylation process catalyzed by acetyltransferase in which an acetyl group from acetyl-CoA is covalently transferred to the ε-amino group of lysine residues (typically) within the target substrates (32, 33). Deacetylase enzymes can also reverse this biochemical process. Acetylation is critical in chromatin remodeling, gene expression, and protein function regulation (34). Many nonhistone proteins, like histones, are sensitive to acetylation and other PTMs (35). Because lysine acetylation was first discovered in histones, many KATs and KDACs are also known as histone acetyltransferases (HATs) and deacetylases (HDACs), respectively (36). Aberrant acetylation of nonhistone proteins is common in several human disorders, particularly cancer (37). Acetylation is also closely associated to active glycolysis in tumors. Phosphoglycerate kinases that include PGK1 and PGK2 are ATP producing glycolytic enzymes and few reports have showed acetylation at K323 in PGK1 increases its enzyme activity and glucose absorption, causing an increase in aberrant hepatic cell growth and carcinogenesis (38). Enolase 2 (ENO2) is another important glycolytic enzyme that is overexpressed in prostate cancer, small-cell lung cancer, metastatic neuroblastoma, and leukemia (39, 40).

### Glycosylation

Glycosylation is the most common and diversified type of protein post-translational modifications that is vital for many biological processes (41). Glycosylation is an enzymatic process by which saccharides form glycosidic bridges with other saccharides, proteins, or lipids (42, 43). Protein glycosylation are of two types: N-linked glycosylation (N-glycosylation) and O-linked glycosylation (O-glycosylation), with O-linked being a more difficult modification due to its structural complexity and diversity (41). Different glycoconjugates disrupt essential cancer cell functions as well as the tumor microenvironment, resulting in carcinogenesis. Mostly, cancer cells diverge from the classical glycosylation pathway, resulting in aberrant glycan expression (44). O-GlcNAc modifications have been linked to tumor cell proliferation employing FoxM1, a transcription factor and cyclin D1, both of which are involved in cell cycle progression, cancer cell survival, and angiogenesis (45).

### Methylation

Methylation is a classic post-translational modification utilized to transfer information in signal transduction pathways in the cell. Protein methylation can occur at both their N- and C-termini, as well as on side chain nitrogen atoms of lysine and arginine residues (46, 47), mediated by protein methyltransferases (PRMTs) (48). PRMTs, which are a family of enzymes that methylate both histone and nonhistone proteins (49) are of two types, namely, lysine methyltransferases (KMTs) and protein arginine methyltransferases (RMTs). Lysine residues can be mono-, di-, or trimethylated, but arginine residues can only be mono- or can remain demethylated (50). PRMT family members were identified to be overexpressed in a variety of cancers (51, 52). Elevated PRMT3 expression has been linked to poor clinical outcomes in pancreatic cancer (53), colorectal cancer (54), and hepatocellular carcinoma (55). In lung adenocarcinoma, the tumor suppressor DAL-1/4.1B interacts with PRMT3 and suppresses its methyltransferase activity, signifying a putative involvement of PRMT3 regulation in tumor progression (56).

### Sumoylation

SUMOylation is an important post-translational protein modification that covalently attaches small ubiquitin-like modifiers (SUMO) in the ϵ-amino group of lysine residues in the target protein, via a multi-enzymatic reaction. SUMO is an 11 kDa protein which has a structural similarity with ubiquitin (57). SUMO is covalently linked to a lysine residue in the substrate protein via three enzymes: activating (SUMO E1), conjugating (SUMO E2), and ligase (SUMO E3) (58). Furthermore, SUMO is dissociated from the target protein by SUMO proteases (sentrin-specific protease 1, SENP1 in humans) (59). The enzymes involved in SUMO pathway are commonly increased in numerous malignancies and have been associated to carcinogenesis and poor patient prognosis. SUMOylation has been linked to chemoresistance and hormone therapy resistance in many studies. Also, the regulation of the oncogene Myc and the SUMOylation machinery has been discovered in pancreatic cancer (60).

### Ubiquitination

Ubiquitin (Ub), a 76-amino acid regulatory protein, is covalently attached to protein substrates using a series of enzymatic cascade that involves Ub-activating (E1), Ub-conjugating (E2), and Ub-ligating (E3) enzymes in a highly conserved post-translational modification called as Ubiquitination (or, Ubiquitylation). Ub attaches to substrates in multiple ways, such as a single Ub attached to an amino acid (monoubiquitination) or to many amino acids (multiple monoubiquitination), or as polymeric chains (polyubiquitination). Different Ub chains are produced via isopeptide linkage with the N-terminal methionine (M1) and seven internal lysine (K) residues (61). As a result, mono- or polyubiquitination affects the function of several proteins under healthy and/or pathological events (62, 63). Deubiquitinases (DUBs) inhibit ubiquitination by eliminating Ub modifications from their target substrates, which are crucial for cell cycle, apoptosis, and gene transcription (64, 65). Abnormal ubiquitination may contribute to tumor growth and tumorigenesis (66). Mutations in E3 ligases can rapidly degrade tumor suppressors or, conversely, loss of ubiquitination in growth-promoting oncoproteins (67). Furthermore, E3 ligases and counterbalancing Deubiquitinases are interesting prospective targets for cancer therapies because of their substrate specificity.

### Neddylation

Neddylation is a reversible covalent attachment of a ubiquitin-like molecule NEDD8 (neuronal precursor cell-expressed developmentally down-regulated protein 8) to a lysine residue of the protein substrate (68, 69). The NEDD8-activating enzyme E1, the NEDD8-conjugating enzyme E2, and substrate-specific NEDD8-E3 ligases all work together to promote neddylation, similar to ubiquitination (70–72). The key family of multiunit E3 ubiquitin ligases, Cullin-RING ligases (CRLs), regulate 20% of proteasome-mediated protein degradation (73–76). Targeting cullin neddylation looks to be an appealing cancer therapeutic strategy, provided hyperactivation of CRLs support carcinogenesis (77, 78). Aberrant activation of the neddylation pathway results in increased global neddylation of substrates such as cullins, leading to subsequent degradation of tumor suppressors (e.g., p21 and p27) and facilitating carcinogenesis and progression (71, 79). Thus, utilizing the neddylation route to inactivate CRLs can be a feasible anticancer approach.

### S-Nitrosylation

S-nitrosylation is a covalent post-translational modification in which a nitric oxide (NO) molecule is coupled to the reactive thiol of an adjacent cysteine residue to generate S-nitrosothiol (SNO) (80). Endogenous NO is synthesized from L-arginine in mammalian cells by three distinct gene-encoded NO synthases (NOSs): neuronal NOS (nNOS/NOS-1), inducible NOS (iNOS/NOS-2), and endothelial NOS (eNOS/NOS-3) (81). S-nitrosylation, like other post-translational modifications, regulates biological activity of a wide range of proteins *in vivo*, as well as controls transcription (82), DNA repair (83), and apoptosis (84–86). Aberrant S-nitrosylation has been associated with significant pathophysiological events, including reported studies that influence the initiation of cancer and cancer cell proliferation both directly and indirectly (83, 87). Bentz et al. demonstrated in 2000 that nitrotyrosine levels are elevated in reactive and dysplastic types of head and neck squamous cell carcinoma (HNSCC) lesions than in normal squamous mucosa (88).

### Succination

Succination is a stable post-translational modification that occurs when the Krebs cycle intermediate fumarate combines with the thiol groups on cysteine residues to produce S-(2-succino)cysteine (2SC) (16). Some studies have found that 2SC is a biomarker for not only mitochondrial stress in obesity and diabetes, but also fumarate hydratase (FH) deficiency in individuals with hereditary leiomyomatosis and renal cell carcinoma (HLRCC) (16, 19, 89, 90). Interestingly, fumarate can enter other cellular and extracellular compartments, most likely because fumarate can be transported from mitochondria via specialized carriers, causing 2SC production and extra-mitochondrial protein impairment. The pool of impaired proteins is referred to as the succinated proteome, which then loses its ability to perform functional and structural activities, possibly leading to cell death (91). There is substantial evidence that shows elevated levels of protein succination in diabetes (17, 92), obesity (89), and cancer (93, 94). As a result, 2SC is identified as a mitochondrial stress biomarker, which may result in cellular dysfunction and even apoptosis in cancer and diabetes (91).

### Prenylation

Prenylation is a common covalent post-translational modification that occurs in all eukaryotic cells and is mediated by the enzymes protein farnesyltransferase (FTase) or protein geranylgeranyltransferase I (GGTase I), respectively. Prenylation involves the attachment of either a 15-carbon farnesyl or a 20-carbon geranylgeranyl isoprenoid lipid to the cysteine residues (95). When the farnesyl isoprenoid is involved, this enzyme-catalyzed process is known as farnesylation and when the geranylgeranyl isoprenoid is involved, the reaction is known as geranylgeranylation (96). Prenylation is the first crucial step in membrane targeting and binding, as well as mediating protein-protein interactions for many of these proteins; heterotrimeric G-proteins also depend on prenylation for its function (97). The discovery that protein prenylation was required to maintain the malignant activity of oncogenic Ras GTPases evoked immense interest in this field, as Ras proteins have been known to drive more than 30% of human malignancies (98, 99). Preclinical trials of farnesyltransferase inhibitors (FTIs) in cancer cell lines and animal models were highly effective, which resulted in the entry of four FTIs (as monotherapy or in combination with other anti-cancer drugs) into clinical trials in 2000 (100, 101). This initial victory did not translate into significant anticancer activity in patients with advanced solid tumors or acute myeloid leukaemia (AML), despite promising phase 1 and 2 trials (102). Responses to FTIs, irrespective of cellular or tissue origin, do not appear to rely on RAS mutations, because inhibiting KRAS farnesylation directs to its geranylgeranylation (101). Thus, understanding the molecular mechanism of farnesylated proteins and their inhibition strategies, the therapy regimen for cancer patients responsive to FTI treatment can be augmented.

### Palmitoylation

Palmitoylation is an enzyme-mediated reversible lipid modification by which palmitate, a 16-carbon palmitic acid, is added via a thioester linkage to a cysteine (Cys) residue (103). The enzymes regulating both palmitoylation and depalmitoylation are the DHHC protein family named as palmitoyl acyltransferases (PATs) (104) and enzymes such as the acyl protein thioesterase (APT) and alpha beta hydrolase-domain containing protein 17 (ABHD17) protein family showing depalmitoylase activity (105, 106). Palmitoylation occurs on a number of critical cancer-related proteins. The RAS family of small GTPases is one such classic example (107, 108). A report demonstrated that palmitoylation at Cys181 site is required for oncogenic NRAS connection with the plasma membrane, and palmitoylation removal inactivated numerous signaling pathways downstream of NRAS, thereby inhibiting leukemogenesis (109).

### Succinylation

Succinylation is a post-translational modification that involves the transfer of a succinyl group (-CO-CH_2_-CH_2_-CO_2_H) to a lysine residue via enzymatic or non-enzymatic reactions (110). Succinylation PTM is predominantly mitochondrial of both prokaryotic and eukaryotic organisms (111, 112) and is involved in the production of mitochondrial energy (113, 114). Aberrant succinylation can influence cancer progression, cardiometabolic disorders, hepatometabolic disorders, and neurological disorders (115). Succinylation of a key glycolytic enzyme, pyruvate kinase muscle isotype M2 (PKM2) has been described to increase its enzymatic activity in the context of cancer, but also a different succinylated lysine residue (K498) has been reported, stating that succinylation may have contrasting effects on the same protein target depending on modified site of lysine residues (116).

### Citrullination

Citrullination modification is deamination of a positively charged arginine into the electrically neutral citrulline (a non-coding amino acid), when subjected to an elevated Ca^2+^ concentration (117) catalyzed by the peptidyl-arginine deiminase (PAD). The peptidyl-arginine deiminase (PAD) enzyme family has five isoenzymes (PAD1-4 and PAD6) that target different tissues (118). Vimentin, actin, collagen, fibronectin, keratin, tubulin, and different histone proteins are amongst the proteins found in the human citrullinome (119, 120). A study found that benign tumors and non-tumor inflamed tissues were devoid of PAD4 expression (121), whereas malignant tumors had significantly higher PAD4 levels than similar primary tumors (122), implying that citrullination plays a role in the transformation from benign neoplasm to invasive malignancy.

### S-Glutathionylation

S-glutathionylation is a distinct redox-driven post-translational modification in which the cysteine residue of glutathione forms a disulphide bond with the -SH (thiol) group of a target cysteine in the protein, oxidizing it. The reversible nature of this unique modification can lead to transitory alterations in the target protein’s structure and/or function (123). Few proteins related to cellular metabolism and detoxification are identified to be affected by glutathionylation. One such key metabolic enzyme is pyruvate kinase M2 (PKM2), which is critical for cancer cells due to their reliance on the glycolytic pathway and its potential role in modifying antioxidant responses (124, 125). Anastasiou et al. established that S-glutathionylation of PKM2 Cys358 is important for responding to oxidative stress by permitting PKM2 deactivation (125).

## Novel post-translational modifications

### Monoaminylation

Monoaminylation is a biochemical process in which biogenic monoamines (serotonin, dopamine, histamine, etc.) are covalently linked to glutamine residues within certain protein substrates, by Transglutaminase 2, an enzyme that catalyzes the transamidation reaction of primary amines to glutamine-carboxamides (126). Monoaminylation is important in several aspects of cellular plasticity (in the brain and maybe elsewhere) (127). Because monoaminylation is one of numerous newly found histone PTMs, further studies are ongoing that may have biological significance in terms of carcinogenesis and cancer therapy.

### Crotonylation

Protein crotonylation is one of the newly discovered post-translational modifications that occur primarily on the lysine residue, known as lysine crotonylation (Kcr). Kcr is a conserved modification that is controlled by a number of enzymes and co-enzymes, such as lysine crotonyltransferase (writer), lysine decrotonylase (eraser), specific YEATS proteins (reader), and crotonyl-coenzyme A (donor) (128). Crotonylation was first discovered as a PTM on histone lysines (129). Histone Kcr has been linked to a variety of illnesses, including depression, acute renal damage, HIV latency, and carcinogenesis (130–133). Immunohistochemical staining of tumor specimens with adjacent normal tissues reveals that lysine crotonylation occurs in both the cytoplasm and the nucleus, and that lysine crotonylation level is downregulated in liver cancer, gastric cancer, and renal carcinoma, but upregulated in thyroid, esophageal, colon, pancreatic, and lung cancers (134).

### Propionylation

Propionylation (propionyl; C-3 fatty acid chain) is a novel histone acylation modification where in the propionyl group covalent attaches to the lysine residues of the target protein (Kpr). After its discovery in histone proteins, Kpr was also found to be present in nonhistones (135). Propionyl-CoA, the co-substrate for Kpr modification arises from the breakdown product of cholesterol and odd-chain fatty acid oxidation and branched-chain amino acid catabolism, which thereby provides us an insight that Kpr may have a potential role in cellular metabolism that involves chromatin architecture during fasting and catabolism (136, 137). Not much has been observed about the function of propionylation in cancer development yet but it is well-known that most of the acylation modifications act synergistically in tumor pathogenesis (138).

### Butyrylation

Butyrylation is one of the newly discovered biochemical acylation modifications, observed in both plants and animals, in which the butyryl group covalently alters the amino acid, lysine (Kbu). Kbu occurs in both histone and nonhistone proteins (135). The butyryl group or butyrate (C-4 fatty acid chain) plays an important role in several human diseases, such as vascular dementia, diabetes, psychiatric disorders, and cancer whereas various plant-based substances have also been potentially associated to Kbu of histones (139–141). p300 and CBP were discovered as butyryl-modifying enzymes, that allows histone H4 to get propionylated and butyrylated. Soon after Kbu was identified, SIRT enzymes (Sirt7) with debutyrylase activities were identified that act as erasers (142). A recent study has made a comparative analysis of histone markers in two types of esophageal squamous cell carcinoma using mass spectrometry, and found that various modifications, such as lysine methylation, acetylation, as well as butyrylation on histones H3 and H4 work together in the development of cancer (143). Since these are novel acylation modifications, further studies are required to explore their roles in different disease pathologies.

### Lactylation

Lactylation is another novel post-translational modification that occurs when lactate (L-lactate rather than D-lactate) changes lysine residues on histone proteins (Kla or Klac) (144, 145). Cancer cells rely on aerobic glycolysis to produce energy and metabolites, forming lactate, which supports lysine lactylation (Kla) (145). The role of histone Kla has been investigated in mouse embryo fibroblasts (MEFs), ocular melanoma cells (146), and non-small cell lung cancer (NSCLC) cells (147), suggesting that Kla is important in controlling pluripotency and oncogenesis. Recently it was found that Kla levels were considerably greater in 51 tumor tissues as compared to its corresponding para-cancerous tissues, when pan-Kla levels in gastric tumors and adjacent tissues were evaluated (148). Furthermore, higher Kla levels were associated with poorly differentiated tumors, lymph node metastases, and poorer prognostic rates of patients, thus implying that Kla has prognostic significance in gastric tumors (148).

## Discussion

**Figure 2:**
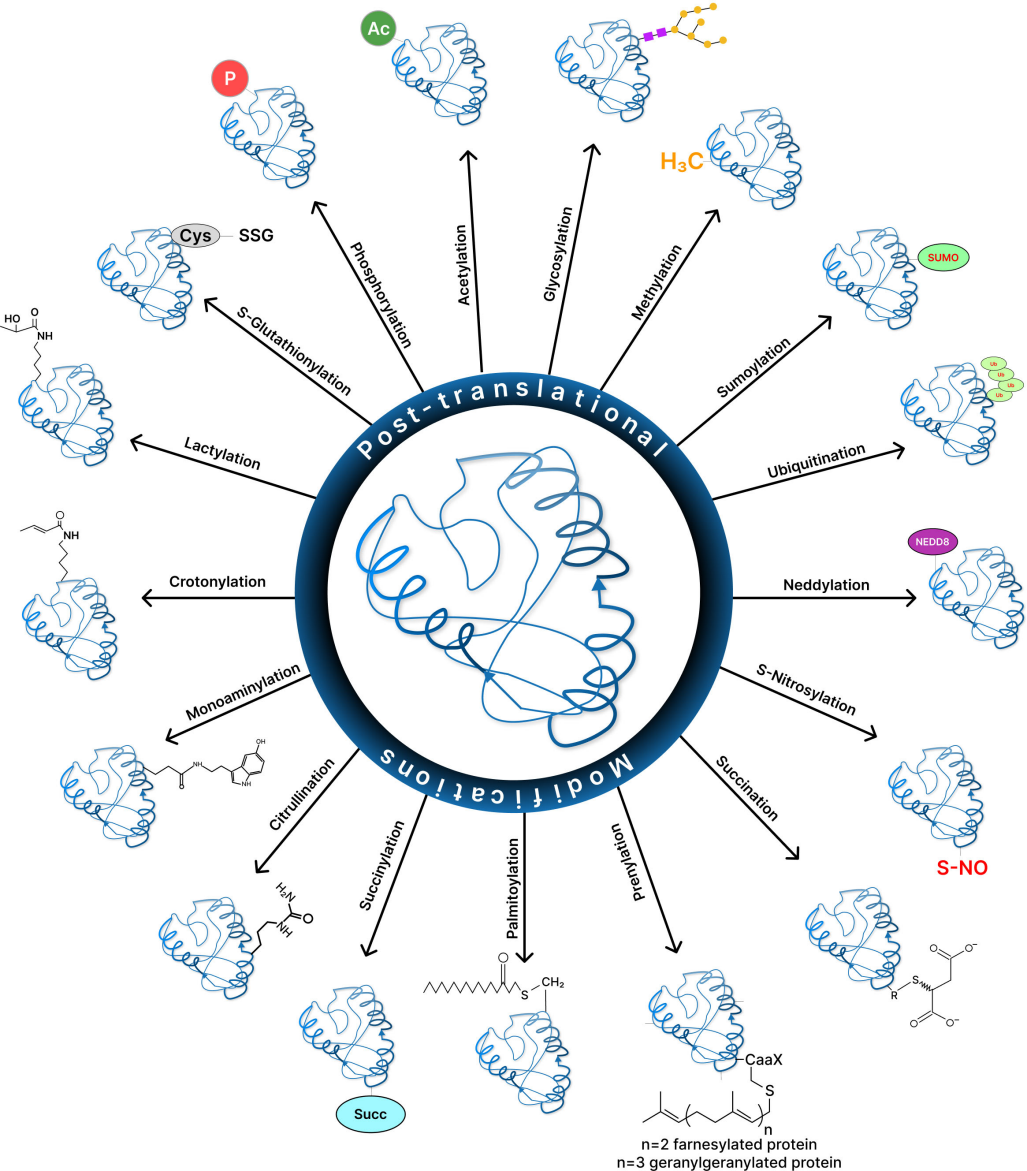
Various post-translational modificalions in cancer.

Post-translational modifications (PTMs) affect many essential biological processes by reversibly altering the conformation, function, and/or interaction regions of a protein. Aberrant changes are signs of cellular stress or malfunction, and they have been linked to cancer and a variety of other diseases. Understanding which proteins has changed, at which locations, and the associated biological consequences are therefore critical (**Figure 2**). Therefore, identification of a multitude of post-translationally modified protein sites has become an important and promising endeavor with the advent of mass spectrometry (MS)-based proteomics, chemical biology tools, fluorescence-based, and bioinformatic techniques (149, 150). However, these cutting-edge approaches present a number of complex challenges. Few of the difficulties may be to determine which proteins are altered, at what locations, and what biological relevance it has. Also, whether cells are biased on non-enzymatic over enzymatic pathway of modification and what is the reason behind that preference can be some of the intriguing questions to consider.

Exciting breakthroughs in identifying PTMs on a proteome-wide and cellular levels show a growing impact in understanding the proteome’s diversity. Once PTM analysis at the proteome level becomes regular, the role of PTMs in disease can be explored considerably more systematically than before and will promote in the discovery of novel drugs and cancer therapeutic strategies. Few key proteins whose post-translational modifications are clinical targets for cancer therapeutics as well as associated with the hallmarks of cancer or its pathophysiology have been enlisted (**Tables 2**, **3**).

**Table 2:** Examples of clinically targeted oncoproteins and the associated post-translational modifications responsible for cancer therapeutics.

Oncoprotein	Description
Signal transducer and activator of transcription 3 (STAT3)	Studies have shown that knockdown of Raf Kinase Inhibitor Protein (RKIP, negative regulator of Ras-MAPK/ERK signaling pathway) in NSCLC patients, causes STAT3 phosphorylation and activation Sleading to metastasis in A549 and NCI-H1299 cell lines (1, 2).
Epidermal Growth Factor Receptor (EGFR)	In NSCLC patients, palmitoylation of EGFR oncoprotein at Cys^797^ site by ZDHHC1, ZDHHC2, and/or ZDHHC21 enzymes (Zinc finger DHHC-type containing enzymes) leads to receptor dimerization, stability, and its subsequent activation (3). When EGFR palmitoylation is inhibited using broad spectrum protein palmitoylation inhibitors such as 2-bromopalmitate (2-BP), the lethal efficacy of its targeted inhibitor, Gefitinib increases (3).
N-acetylgalactosaminyltransferases (GALNTs)	GALNTs are group of enzymes that are responsible at modulating initial mucin-type O-glycosylation, which have shown close relationship with cancer incidence and metastasis (4, 5). Previous animal studies have extensively reported that GALNT class of enzymes (GALNT1-GALNT20) act as prognostic markers for different cancers, or have been linked to alterations in cellular characteristics including cell proliferation, migration, invasion and metastasis in experimental models (6). Few of the broad range of cancers in which altered protein levels of GALNTs have been demonstrated include colorectal (7), gastric (8), breast (9), pancreatic (10), lung adenocarcinoma (11), NSCLC (12), and glioma (13). Studies showed that GALNT2 promotes tumor formation and progression in BALB/c nude mice models (14). Many transmembrane proteins are modified by N-linked glycosylation, which is responsible for tumor metastasis. This study also demonstrates that N-glycosylation of T-cell membrane protein-4 (TIM-4) at Asn^291^ site is conducive to cancer metastasis as reported *in vitro* and *in vivo* models (15).
RAS	The RAS family of GTPases are small monomeric G-proteins that regulate key biological functions like gene expression, transmembrane signaling, apoptosis, etc (16). RAS has been extensively studied and play significant roles in cancer initiation and progression. About 25-30% of human tumors harbor RAS mutations. KRAS, NRAS, HRAS are three members of this oncogene superfamily that have been reported to implicate in various malignancies; KRAS in NSCLC, pancreatic cancer; NRAS in liver cancer and in liquid tumors like AML; HRAS mutations in BCa and renal cell cancer (17–19). HRAS oncogene is only **farnesylated**, but KRAS4A, KRAS4B and NRAS are **geranylgeranylated** by GGTaseI, leading to activation of downstream signaling of MAPK/ERK pathway (20). Because many tumors rely on RAS oncogenes for their survival, a number of drugs have been developed that selectively target these oncoproteins. Earlier, researchers focused on blocking a type of **prenylation** PTM known as farnesylation on RAS which exhibited huge promise in preclinical investigations (19). Unfortunately, no therapeutic effects from farnesyl transferase inhibitors could be demonstrated, making RAS challenging to drug, but inhibitors targeting specific RAS mutations are under development (21).
MYC (*c-MYC/N-MYC/L-MYC)*	MYC family of transcription factors has several roles to play in cellular processes such as cell growth, proliferation, differentiation, and programmed cell death (22). It is deregulated in ~ 70% of human tumors, of which majority are aggressive such as in leukemias and high-grade lymphomas or in cancers which have poorer clinical outcomes like NSCLC (23). Studies have showed that the compound oridonin (a natural terpenoid) upregulates SCF^FBXW7^ causing **degradation** of MYC protein via Ub-proteasome system, and therefore leading to apoptosis in leukemia and lymphoma cell lines (24). Aurora kinase A (AURKA) blocks SCF^FBXW7^-mediated MYC proteolysis by binding MBI motif based on a covalent association that exclusively depends on the **phosphorylation** status of T^58^ and S^62^ sites (25, 26). Overall, strategies are being made to develop small molecules that can inhibit AURKA to indirectly target MYC activity. Drugs such as MLN8237/alisertib and CD532 causes Aurora A-c-MYC/N-MYC complex non-functional and advances the tumor towards regression, as observed in neuroblastoma and human hepatocarcinoma cells (26, 27).

**Table 3:** Examples of key proteins with its corresponding post-translational modification(s) (in bold) related to cancer hallmarks/responsible for cancer pathophysiology.

Protein Name	Type of PTM(s) involved
Programmed Death-Ligand 1 (PD-L1/*CD274*)	Epigenetic modifications, such as **histone acetylation** and **methylation** of H3K3me3. - Histone acetylation helps BET domain proteins (BRD4) to associate with PD-L1. Also, HDAC inhibitors augment histone acetylation and upregulate PD-L1 expression in tumors (28–30). - Trimethylation of histone H3 on lysine 4 increases PD-L1 expression in cancer cells. For example, Histone methyltransferase (HMTase) MLL1 associate with *CD274* promoter and catalyzes H3K4me3, thereby upregulating its expression, which in turn boosts PD-L1 mRNA levels in tumors (31).
N-acetylgalactosaminyltransferases (GALNTs)	GALNTs are group of enzymes that are responsible at modulating initial mucin-type O-**glycosylation**, which have shown close relationship with cancer incidence and metastasis (4, 5). Previous animal studies have extensively reported that GALNT class of enzymes (GALNT1-GALNT20) act as prognostic markers for different cancers, or have been linked to alterations in cellular characteristics including cell proliferation, migration, invasion and metastasis in experimental models (6). Few of the broad range of cancers in which altered protein levels of GALNTs have been demonstrated include colorectal (7), gastric (8), breast (9), pancreatic (10), lung adenocarcinoma (11), NSCLC (12), and glioma (13). Studies showed that GALNT2 promotes tumor formation and progression in BALB/c nude mice models (14). Many transmembrane proteins are modified by N-linked glycosylation, which is responsible for tumor metastasis. This study also demonstrates that N-glycosylation of T-cell membrane protein-4 (TIM-4) at Asn^291^ site is conducive to cancer metastasis as reported *in vitro* and *in vivo* models (15).
Prohibitin (PHB)	PHBs (PHB1 and PHB2) are evolutionary conserved scaffold proteins that are ubiquitously expressed in mitochondria, plasma membrane and nucleus. They exhibit diverse compartment-specific regulatory roles such as cell proliferation, metabolism, lipid scaffolding in cell membrane, mitochondrial remodeling, and apoptosis (32–35). PHBs play an important role in carcinogenesis by regulating vital cellular signaling pathways (such as the Ras-Raf-MEK-ERK signaling axis) that leads to cell proliferation, and metastasis and these proteins are tightly controlled by **phosphorylation (** 36, 37). In bladder cancer (BCa), the expression levels of PHB1 were found to be higher (in 141 out of 167 patients), indicating that PHB1 is required for BCa cell proliferation (38). Another report demonstrated that mitochondrial localization of PHB is regulated by Protein Kinase B (PKB)/Akt-mediated phosphorylation of Prohibitin at Thr^258^ site (in the cytoplasm) in BCa patients. Thus, phosphorylated-PHB is a suitable therapeutic target in BCa (39). Besides, phosphorylation of PHB1 at Ser^121^, Tyr^259^, and Tyr^114^ regions is also responsible for tumor formation (40, 41).
MYC (*c-MYC/N-MYC/L-MYC)*	The oncoprotein MYC is a pleiotropic transcription factor that affects essential cellular functions such as proliferation, differentiation, cell cycle, metabolism, and apoptosis (23). Strong evidence defines both aberrant MYC expression as an oncogenic driver of malignancies as well as its association to all of cancer ‘hallmarks’ (42, 43). Surprisingly, MYC allows tumor cells to escape immune-surveillance either by downregulating MHCI expression or by upregulating inhibitory cytokines and immune-checkpoint proteins such as PD-L1 and CD47, thereby shedding light on associating MYC inhibition and escape of immune-checkpoints to target MYC (44–46).
Fructose-1,6-bisphosphatase (FBP-1)	Fructose-1,6-bisphosphatase (FBP-1 or FBPase) is a rate-limiting enzyme catalyzing the conversion of fructose 1,6-bisphosphate to fructose 6-phosphate and inorganic phosphate, which is an important step in gluconeogenesis, required for the regulation of energy metabolism and maintenance of glucose homeostasis (47). Evidences support that FBP-1 downregulation is considered to be as a prognostic cancer marker for different tumor types such as gastric cancer, lung cancer and breast cancer, increasing the glycolytic rate and cancer stem cells in number (48–50). Recent reports demonstrated that TRIM28, which is an E3 Ubiquitin ligase, helps in regulation of FBP-1 expression levels by some post-translational changes in hepatic cancer. Furthermore, it was observed that TRIM28 directly associate with and recruits to **ubiquitination** by degrading FBP1 protein. Direct interacting partners of TRIM28 such as MAGE-A3 and MAGE-C2 (51, 52), can augment TRIM28-specific degradation of FBP1 by forming a ternary ubiquitin ligase complexes with TRIM28 (53).
Heat shock protein 90 (HSP90)	HSP90 is an evolutionarily conserved molecular chaperone that participates in cellular homeostasis and is an important mediator of oncogene addiction (54, 55). Studies showed that NO-dependent **nitrosylation** at Cys^597^ position in C domain of HSP90 blocks chaperone function in endothelial cell lines (56). Existing findings about anti-cancerous activity of externally administered NO may have been caused from its blockade of HSP90 in tumors (57), which is consistent with the observations that HSP90 in regulation of telomeric activity (58).

A number of small molecule drugs that target PTM regulators, such as kinase, deacetylase, and methyltransferase inhibitors, have been approved for cancer treatment. However, in addition to the therapeutic target, each upstream regulator often has several substrates. Therefore, unbiased inhibition of PTM regulators may result in off-target effects, limiting their therapeutic usage. As a result, ‘small’ molecules with profound selectivity that can modulate a particular PTM of a protein are in huge demand. Many chemical proximity-induced PTM methods, such as proteolysis-targeting chimera (PROTAC), have arrived in the last two decades as having immense potential for targeting ‘undruggable’ proteins and for clinical translation in the treatment of cancer (151, 152).

## Author contributions

HD and NJ wrote and drafted the manuscript. All authors contributed to the article and approved the submitted version.
